# Interactive web-based pulmonary rehabilitation programme: a randomised controlled feasibility trial

**DOI:** 10.1136/bmjopen-2016-013682

**Published:** 2017-03-31

**Authors:** Emma Chaplin, Stacey Hewitt, Lindsay Apps, John Bankart, Ruth Pulikottil-Jacob, Sally Boyce, Mike Morgan, Johanna Williams, Sally Singh

**Affiliations:** 1 Leicester Respiratory Biomedical Research Unit, Department of Respiratory Medicine, Centre for Exercise and Rehabilitation Science, Glenfield Hospital, University Hospitals of Leicester, Leicester, UK; 2 Department of Primary Care and Health Sciences, Keele University, Keele, UK; 3 Health Sciences Research Institute, Medical School, University of Warwick, Coventry, UK; 4 School of Sport, Exercise and Health Sciences, Loughborough University, Loughborough, UK

**Keywords:** SPACE for COPD, chronic obstructive pulmonary disease, Internet, Web-based, pulmonary rehabilitation

## Abstract

**Objectives:**

The aim of this study was to determine if an interactive web-based pulmonary rehabilitation (PR) programme is a feasible alternative to conventional PR.

**Design:**

Randomised controlled feasibility trial.

**Setting:**

Participants with a diagnosis of chronic obstructive pulmonary disease were recruited from PR assessments, primary care and community rehabilitation programmes. Patients randomised to conventional rehabilitation started the programme according to the standard care at their referred site on the next available date.

**Participants:**

103 patients were recruited to the study and randomised: 52 to conventional rehabilitation (mean (±SD) age 66 (±8) years, Medical Research Council (MRC) 3 (IQR2–4)); 51 to the web arm (mean (±SD) age 66 (±10) years, MRC 3 (IQR2–4)). Participants had to be willing to participate in either arm of the trial, have internet access and be web literate.

**Interventions:**

Patients randomised to the web-based programme worked through the website, exercising and recording their progress as well as reading educational material. Conventional PR consisted of twice weekly, 2 hourly sessions (an hour for exercise training and an hour for education).

**Outcome measures:**

Recruitment rates, eligibility, patient preference and dropout and completion rates for both programmes were collected. Standard outcomes for a PR assessment including measures of exercise capacity and quality of life questionnaires were also evaluated.

**Results:**

A statistically significant improvement (p≤0.01) was observed within each group in the endurance shuttle walk test (WEB: mean change 189±211.1; PR classes: mean change 184.5±247.4 s) and Chronic Respiratory disease Questionnaire-Dyspnoea (CRQ-D; WEB: mean change 0.7±1.2; PR classes: mean change 0.8±1.0). However, there were no significant differences between the groups in any outcome. Dropout rates were higher in the web-based programme (57% vs 23%).

**Conclusions:**

An interactive web-based PR programme is feasible and acceptable when compared with conventional PR. Future trials maybe around choice-based PR programmes for select patients enabling stratification of patient care.

**Trial registration number:**

ISRCTN03142263; Results.

**Strengths and limitations of this study**

- The study concentrates on the feasibility of an interactive web-based pulmonary rehabilitation (PR) programme (SPACE: Self-management Program of Activity, Coping and Education for chronic obstructive pulmonary disease).
- It provides data on recruitment, eligibility and patient preference which will inform future trials around choice-based programmes for select patients.
- A limitation to the study was a lack of engagement despite patient involvement in the site development.
- Limitations were identified when recruiting patients to a technology-based intervention, in that patients needed to be competent users with an in-depth, specific web-based knowledge.
- The study compares a variety of clinical outcomes between a web-based programme and a conventional rehabilitation PR programme. This facilitates a personalised approach to rehabilitation.

## Introduction

Chronic obstructive pulmonary disease (COPD) is the fourth leading cause of death in the UK and is characterised by a progressive deterioration of debilitating symptoms and increasingly frequent exacerbations. Pulmonary rehabilitation (PR) has been proven to be effective in improving quality of life, psychological functioning and physical activity, and national guidelines recommend that PR should be offered and made available to all those with COPD.^1^ The standard provision of PR is a supervised package of exercise and education usually twice a week for a minimum of 6 weeks, which is either hospital or community based, and supported by a home exercise programme.^2^ However, the barriers to uptake of a PR programme have previously been reported^3^ which included transport, the perceived benefits of PR, disruption to usual routine and the timings of programmes. These factors play some contribution as to why programmes have poor attendance and adherence resulting in many of the programmes reporting dropout rates as high as 50%.

Ongoing changes and challenges means that the National Health Service (NHS) and the services it provides need to adapt to take advantage of and capitalise on the opportunities that new technologies and treatments can offer to patients.^4^ There is a growing evidence base for the use of the internet in the management of many chronic conditions in areas as diverse as the management of diabetes, Parkinson's disease, depression, rheumatoid arthritis, asthma, chronic pain and epilepsy.^5–11^ Computer-tailored interventions have been shown to effectively improve health behaviours such as physical activity^12^ and be cost-effective.^13^ The provision of a menu-based and patient-centred service is said to be essential to improve uptake and completion rates within cardiac rehabilitation (CR).^14^ However, at present there is no choice within PR. A web-based PR programme has the potential to be a novel and effective approach to increasing patient choice in the mode of delivery and setting of rehabilitation (especially to those patients who decline the offer of conventional PR) while simultaneously increasing the capacity of PR.

We have previously developed and described ‘Active Your Heart’ (AYH)^15^ ^16^ which is an interactive web-based CR programme that has proved to be very popular with patients. Brough *et al*^15^ reported a significant improvement in exercise capacity and quality of life in patients that completed the web-based programme. Following the success of AYH, we have developed a prototype website based on the educational content of the ‘SPACE for COPD’ self-management workbook. SPACE (Self-management Programme of Activity, Coping and Education) for COPD^17^ is a structured programme of exercise, education and psychosocial support which has been developed by our institution as a collaboration between experts, patients and carers and has been awarded a Crystal Mark for Clarity by the Plain English Campaign.^18^

The aim of this feasibility study was to provide quantitative, economic and technical data to see if an interactive web-based PR programme was a feasible alternative compared with conventional PR. This included:

1. Gathering information regarding the recruitment rate of patients who were eligible and willing to be randomised to either the web-based programme or the conventional rehabilitation programme, and to monitor retention and dropout through all stages of the programmes.
2. Comparing a variety of clinical outcomes between a web-based programme and a conventional rehabilitation PR programme in order to test out the various components of the intervention and identify any technical or other difficulties that may be inherent in the delivery of a web-based PR programme.

## Methods

### Participants

#### Eligibility criteria for participants

Eligible participants had an established diagnosis of COPD defined as a forced expiratory volume in 1 s (FEV_1_), postbronchodilation of <80% and a predicted ratio of FEV_1_ to forced vital capacity of 0.70 and a Medical Research Council (MRC) dyspnoea score^19^ of between 2 and 5. Patients had to be willing to partake in either arm of the study. Access to the internet for more than 3 months, the ability to navigate around a variety of websites (eg, uses online shopping or banking websites) and regular use of email was required. Patients also had to be able to read and write in English.

Patients were excluded if they were unable to participate in the exercise component of the rehabilitation programme due to other comorbidities or had done PR in the previous 12 months. Eligible patients had to be willing and able to take part in the web-based programme.

#### Setting

Participants to the study were primarily recruited from those patients that had been referred for PR at University Hospitals of Leicester (UHL) NHS Trust. Recruitment was also directly from primary care and community rehabilitation services within Leicester Partnership Trust (LPT) and eligible participants were identified from the research participant database of the Leicester Respiratory Biomedical Research Unit and Pulmonary Rehabilitation Department.

### Randomisation

Patients were randomised to either the conventional rehabilitation programme as is standard at their referred site or the web-based PR programme (SPACE for COPD). Randomisation to the treatment group allocation was on a 1:1 ratio to either group and was performed using a web-based programme (http://www.sealedenvelope.com).

### Trial interventions

#### Intervention group—web-based PR programme

Following randomisation to the intervention group, patients attended a standardised introductory session where participants were given a password-protected secure log-in to the website as well as written instructions on website navigation.

**Figure 1:**
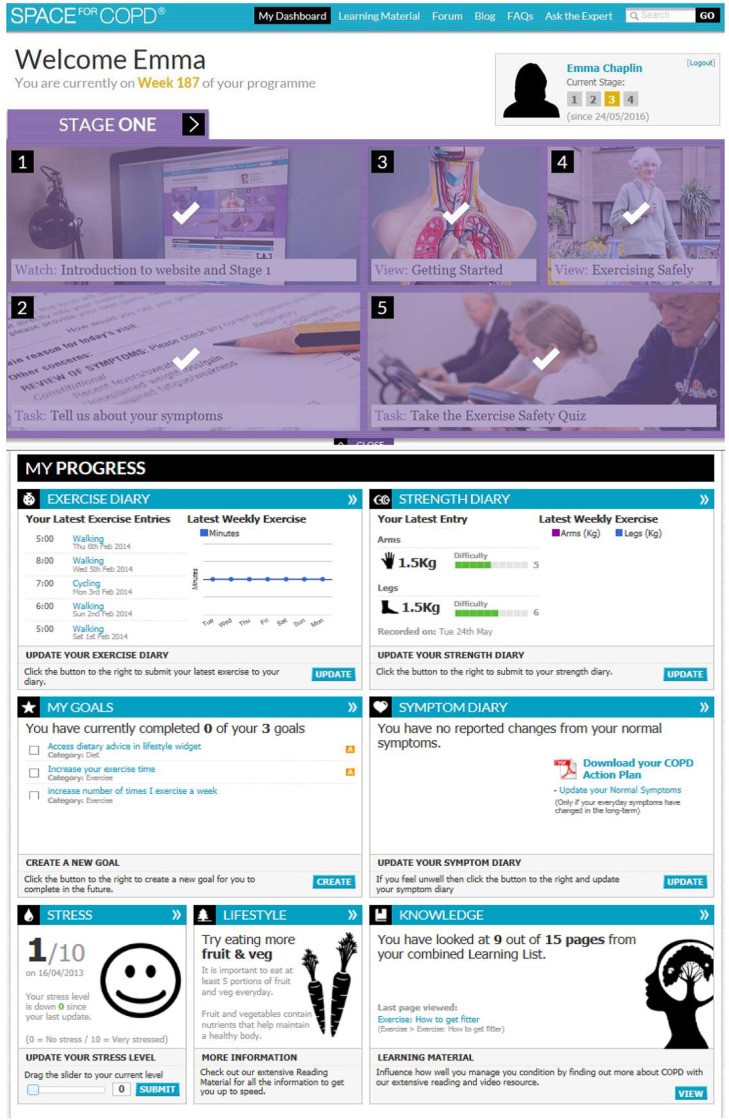
SPACE for COPD dashboard screen showing tasks completed in stage 1 as well as an overview of exercise progression, goals, knowledge and symptom diary. COPD, chronic obstructive pulmonary disease; SPACE, Self-management Program of Activity, Coping and Education.

Patients were directed to all the relevant sections on the website including the home exercise programme and goal setting. There was also an individualised webpage (figure 1) featuring a personalised action plan designed to assist in the management of exacerbations which was completed by the rehabilitation specialist in conjunction with the patient.

As in conventional PR, patients were encouraged to exercise on a daily basis at home and record their progress in the online exercise diary section. The exercise programme consisted of aerobic and strength training. The intensity of the walking was based on their performance on the baseline maximal shuttle walking exercise tests and prescribed at 85% of baseline performance. An exercise target was set by the patient to achieve and work towards each week. Strength training consisted of upper and lower limb resistance training with hand-held weights. Patients recorded how difficult they found the walking and strength training using a visual analogue scale (VAS). Both the walking time and strength were progressed maintaining a VAS rating of 4–7. Throughout the duration of the web-based programme the patient's progress was reviewed online and there was weekly contact between the patient and the rehabilitation specialist via email or telephone using a standardised proforma. Motivational interviewing techniques were used by the healthcare professional to ensure that patients were helped to progress their exercise programme in the aerobic and strength training appropriately and to answer any queries that arose.

The educational content of the web-based programme was based on the ‘SPACE for COPD’ manual. Patients worked through the website content at their own pace; however, certain milestones needed to be completed or achieved before further content could be accessed in order to ensure appropriate progress through the programme (see online supplementary material for description of the different stages on the website). It was anticipated from previous work^14^ that it would take ∼6–8 weeks to work through the online programme.

10.1136/bmjopen-2016-013682.supp1 supplementary material

#### Standard care group—conventional PR programme

Patients randomised to standard care started conventional rehabilitation according to the standard care at their referred site which was either hospital or community based. The hospital programme consisted of 7 weeks (4 weeks supervised; 3 weeks unsupervised) in total. Patients were advised to not attend if they were having an exacerbation. Any sessions missed could be completed later due to it being a rolling programme. In the community-based programmes, patients could attend a maximum of 12 sessions within the closed programme.

Conventional PR programmes at either referral site consisted of twice weekly sessions each lasting 2 hours which were divided into an hour for exercise training and an hour for an education session covering a variety of relevant self-management topics. The exercise training consisted of aerobic and resistance training. A training walking speed was prescribed from the incremental (ISWT)^20^ and endurance (ESWT)^21^ shuttle walk tests performed at baseline. Walking time was progressed maintaining a moderate-to-severe breathlessness as defined by the BORG dyspnoea scale.^22^ Patients were instructed to walk daily at their PR class training speed. Strength training consisted of upper and lower limb resistance training with dumbbells which was based on 1 repetition maximum. Progression was achieved by maintaining a BORG perceived exertion^23^ rating of 13–15. Static cycling was completed, if tolerated, and intensity was prescribed on the basis of the patient's breathlessness and perceived exertion symptom scores. Patients were encouraged to also complete a home exercise programme on the days when they did not attend rehabilitation classes and to fill in an exercise diary. This enabled the patients' progress to be monitored. The educational sessions were conducted as group sessions and delivered by experts in their field. Topics included medication, relaxation skills, chest clearance and breathlessness management, and energy conservation.

### Outcome measures

All the measures used and collected in the trial including clinical (ISWT and ESWT; Chronic Respiratory disease Questionnaire-Self-Reported (CRQ-SR),^24^ Hospital Anxiety and Depression Scale (HADS),^25^ COPD Assessment Tool (CAT),^26^ PR Adapted Index of Self-Efficacy (PRAISE),^27^ Bristol COPD Knowledge Questionnaire (BCKQ),^28^ Euro-QOL (EQ-5D-5L),^29^ patient cost questionnaire^30^) and non-clinical have previously been described in the study protocol.^31^ Clinical measures were performed at baseline and repeated again at the discharge assessment following completion of either rehabilitation programme (usually ∼6–7 weeks after starting the programme) and were conducted by a research physiotherapist who was blinded to treatment group allocation. Patients were classed as a completer if they had reached stage 3 or above of the web programme, achieving 75% of the programme which is standard in clinical practice for those attending classes. Those patients randomised to the website were offered conventional PR classes if they felt it would be more beneficial at discharge.

Non-clinical outcomes included a web-usage audit for the internet-based programme, recruitment rates, eligibility and patient preference as well as dropout and completion rates in both treatment groups. Any serious adverse events were reported to the sponsor. A serious adverse event was defined as an acute exacerbation of their COPD that resulted in a hospital admission. In order to assess the patients' ability to exercise safely, an exercise safety quiz was completed online before being able to progress onto stage 2 of the programme which involved exercising. Patients were then monitored online and through the weekly contacts.

Qualitative and physical activity data are to be presented in future publications.

### Quantitative data analysis

Data were entered and stored on a secure web-based system (REDCAP) which has discrepancy management features. Data were then transferred from REDCAP to the Statistical Package for the Social Sciences (SPSS) V.18 (SPSS, Woking, Surrey, UK). The data were checked for normality before baseline characteristics were compared between groups using an independent t-test. Analysis was primarily descriptive, that is, estimation of means and SDs, proportion of patients eligible/willing to participate in the study. A paired t-test was used to compare within-group changes and an independent t-test was used to compare the differences between the two treatment groups in the ISWT, ESWT and Chronic Respiratory disease Questionnaire-Dyspnoea (CRQ-D) at the two different time points.

## Results

**Figure 2:**
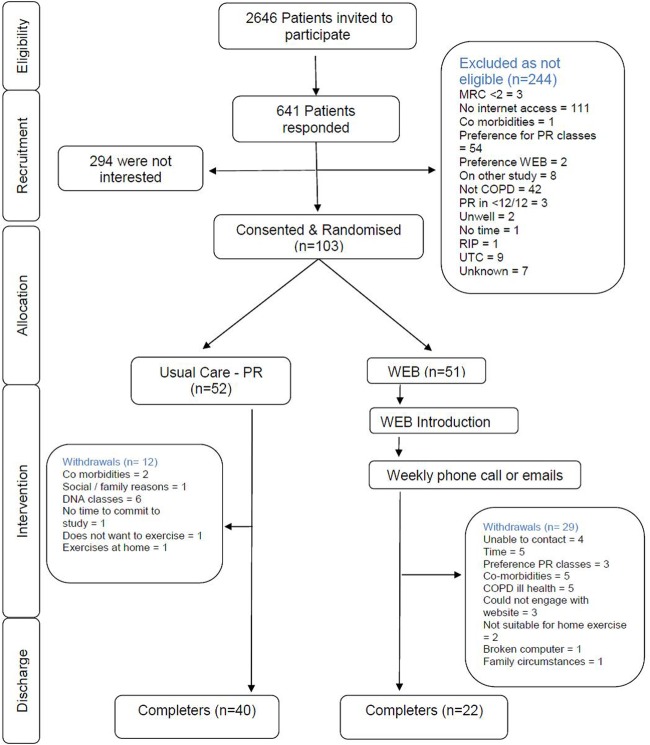
Consolidation standards of reporting trials flow diagram of participation. COPD, chronic obstructive pulmonary disease; MRC, Medical Research Council; PR, pulmonary rehabilitation.

One hundred and three patients were recruited and randomised to the study between May 2013 and July 2015: 52 to the conventional PR group and 51 to the web group. Figure 2 shows the flow of eligibility, screening, randomisation and follow-up in the study. No significant differences between the groups' baseline characteristics or outcome measures (table 1) were seen. More patients dropped out from the web intervention group (n=29) but there were no significant differences between the baseline characteristics of those patients that dropped out of the two groups. Reasons for dropouts are listed in figure 2. The only significant characteristic between web completers and dropouts was the pre anxiety scores (p<0.05) with those that dropped out being more anxious (table 2).

**Table 1:** Baseline characteristics

	PR (n=52)	WEB (n=51)
Age (years)	66.1±8.1	66.4±10.1
Gender (% male)	63.5	74.5
FEV_1_ (% predicted)	55.0±20.5	58.7±29.1
BMI (kg/m^2^)	29.3±6.3	27.9±6.4
MRC (IQR)	3 (2–4)	3 (2–4)
MRC (n)		
2	21	14
3	13	20
4	14	12
5	1	3
Baseline ISWT (m)	284.2±156.0	296.7±180.8
Baseline ESWT (s)	246.2±144.0	241.7±209.7
Pre-CRQ SR-D	2.7±1.1	2.7±1.2
Pre-CAT	20.8±7.5	20.8±8.6
Pre-PRAISE	45.7±7.7	45.6±7.7
Pre-HADS		
Anxiety	7.1±5.0	7.9±4.8
Depression	5.8±3.6	6.4±3.8
Pre-BCKQ	37.1±12.5	33.9±8.6

Data are presented as n or mean±SD.

BCKQ, Bristol COPD Knowledge Questionnaire; BMI, body mass index; CAT, COPD Assessment Tool; COPD, chronic obstructive pulmonary disease; CRQ-SR-D, Chronic Respiratory disease Questionnaire-Self-Report-Dyspnoea; ESWT, endurance shuttle walk test; FEV_1_, forced expiratory volume in 1 s; HADS, Hospital Anxiety and Depression Scale; ISWT, incremental shuttle walk test; MRC, Medical Research Council; PR, pulmonary rehabilitation; PRAISE, PR Adapted Index of Self-Efficacy.

**Table 2:** Baseline characteristics between WEB completers and dropout

	Dropouts (n=29)	Completers (n=22)
Gender (M:F)	18:11	20:2
Age (years)	65.3±12	67.6±7
FEV_1_ (% predicted)	63.6±30.2	52.1±27.2
BMI (kg/m^2^)	29.1±6.7	26.4±5.7
MRC	52% MRC 3	41% MRC 2 27% MRC 3 and 4 4% MRC 5
Pre-ISWT (m)	264.6	334.5
Pre-ESWT (s)	209.1	278.7
Pre-CRQ-D	2.6	2.9
Pre anxiety	9.4*	6.5

*p<0.05 between groups.

BMI, body mass index; CRQ-D, Chronic Respiratory disease Questionnaire-Dyspnoea; ESWT, endurance shuttle walk test; F, female; FEV_1_, forced expiratory volume in 1 s; ISWT, incremental shuttle walk test; M, male; MRC, Medical Research Council.

### Clinical outcome measures

**Figure 3:**
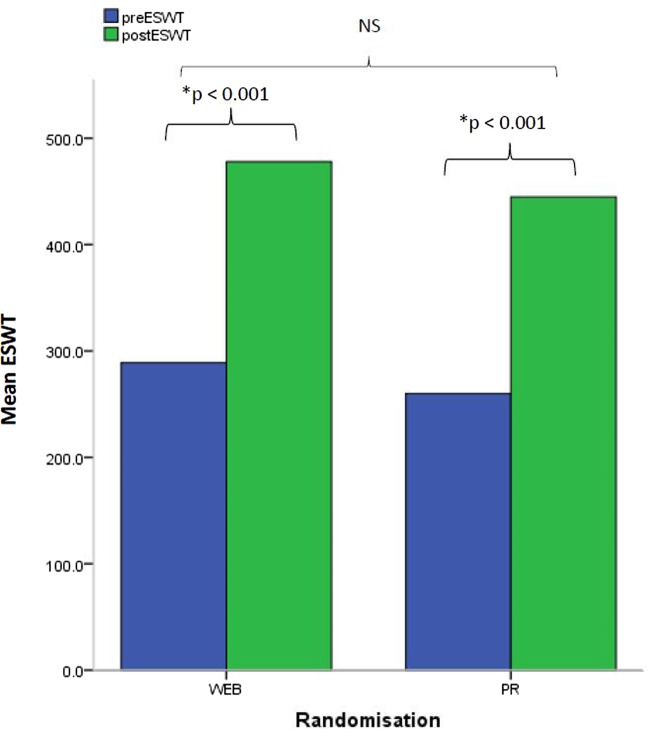
Exercise capacity. Within-group and between-group changes of the ESWT. ESWT, endurance shuttle walk test; PR, pulmonary rehabilitation.

**Figure 4:**
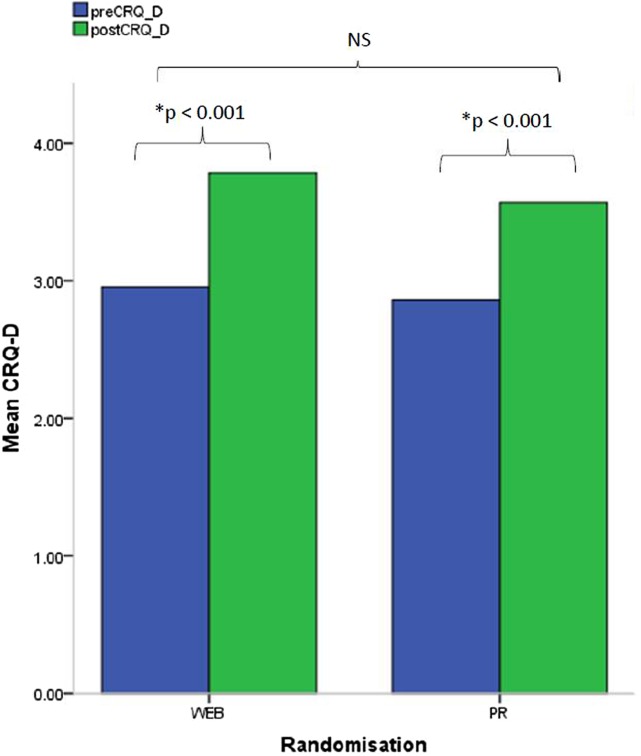
Quality of life. Within-group and between-group changes of CRQ-D. CRQ-D, Chronic Respiratory disease Questionnaire-Dyspnoea; PR, pulmonary rehabilitation.

A statistically significant improvement (p≤0.01) was observed within each group in the ESWT (WEB: mean change 189±211.1; PR classes: mean change 184.5±247.4 s; figure 3) and CRQ-D (WEB: mean change 0.7±1.2; PR classes: mean change 0.8±1.0; figure 4). There were no significant differences between the groups in any clinical outcome. All outcome measures used were feasible to administer.

### Non-clinical study outcomes

The average number of weeks to complete the website was 11±4 with an average number of four logins per week. Patients tended to spend the longest time in stage 2. This was where the exercise programme was started and had the most activities to complete before further content could be accessed. The education material was tailored to the gaps in the patient's knowledge based on those identified from the BCKQ completed at registration and therefore time was spent in different areas accordingly. The stage at which patients dropped out at are listed in table 3. The majority of patients dropped out at the beginning of the web programme which suggests once the patient was engaged with the programme, they were able to complete it. Those that dropped out tended to be mostly MRC 3, had a lower baseline ISWT and a significantly higher HADS anxiety score at baseline compared with those that completed the web programme (table 2). In those patients who had been randomised to the PR classes, the main reasons that they were withdrawn, were the patients not starting the classes and being unable to contact the patient (n=6). Twenty-five per cent of the web withdrawals would have preferred to have attended the classes compared with 54% of patients that attended conventional PR classes preferring to have done the web programme.

**Table 3:** Dropout stages of the WEB programme

Stage	Number of participants
No WEB introduction completed	5
Not registered	7
*Stage 1* Introduction to exercising and goal setting, exercise safety quiz, read educational material	4
*Stage 2* Introduction of aerobic exercise programme, set walking target, read educational material	11
*Stage 3* Introduction of strength training programme, set strength target, continuation of aerobic training and read education material	2
*Stage 4* Maintain strength and aerobic training, review educational material, knowledge quiz	0

**Figure 5:**
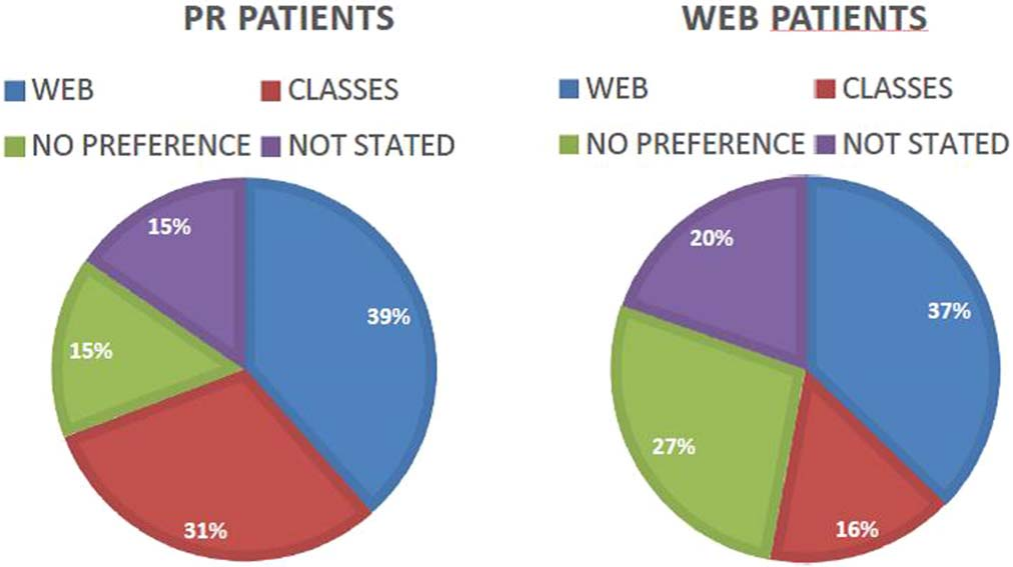
Patient preference for programme setting prior to randomisation.

When patients were asked their treatment preference prior to being randomised, the largest proportion of patients wanted the web programme (n=38%; figure 5). Of the 22 patients that completed the web programme, only 3 patients (n=14%) felt they would like to attend the PR classes.

## Discussion

PR is one of the most effective non-pharmaceutical therapies for patients with COPD, which offers long-term benefits. Issues such as transport and locality still persist for many patients some of which could be addressed by improving accessibility. Data from a recent national COPD audit^32^ suggest that the capacity of PR programmes is inadequate to meet the demand or need. Alternative or more flexible provisions of PR programmes therefore need to be considered.

Voncken-Brewster *et al*^33^ tested the effectiveness of a web-based computer-tailored COPD self-management intervention on physical activity and smoking behaviour. There were no statistically significant effects on health-related behavioural or clinical outcomes. They state this may have been attributed to the low exposure to the application and engagement with the programme has been shown to be crucial for the effectiveness of computer-tailored interventions.^34^ ^35^ Another randomised controlled trial by Pinnock *et al*^36^ found telemonitoring to not reduce hospital admission or improve patients' quality of life. The data from this feasibility study aimed to evaluate the application of an exercise intervention as well as promoting self-management.

The data from this study suggest that an interactive web-based programme has the potential to be a feasible and acceptable alternative when compared with conventional PR. Although both groups improved in the ISWT, the change was not significant and did not meet the minimally clinical important difference (MCID) of 48 m.^37^ The baseline ISWT scores in both groups were higher in these patients compared with those seen normally in our clinical service and therefore may account for the small change in the ISWT score. More patients were MRC 2 in the PR group than are normally referred to the PR service. The change in ISWT may also have been affected due to the structure of the PR programme which was 7 weeks (4 weeks supervised; 3 weeks unsupervised) for the majority of the patients and did not meet the British Thoracic Society (BTS) guidelines^2^ of a minimum of 12 supervised sessions. These guidelines were not published until after the trial had started. Similar improvements in time (3 min) were seen in the ESWTs for both groups. Health-related quality of life, measured by the CRQ-SR, appeared to improve significantly, both groups exceeding the MCID for the CRQ-D of 0.5. High dropout rates in the web arm of the study may have influenced the outcome measures of exercise capacity. The study would suggest, like other rehabilitation studies that using a health-related quality of life measure is a feasible primary outcome measure. However, despite recruiting a large number of participants, the high dropout rate and challenges experienced around a technology-based intervention based on the findings of this study, would potentially make future non-inferiority trials using the CRQ harder; a larger sample size of patients in each group would be required. The use of preference-based randomised controlled trials could be more appealing to patients, therefore improving recruitment and retention rates.

Adherence to web-based programmes can vary due to many reasons ranging from lack of time, to refusing to complete the programme. Several features have been identified that could help to improve adherence to a web-based programme: making the programme tailored to the user and interactive^38^ as well as allowing users to set personal goals.^39^

Initial withdrawals in this study appeared to have been at the exercise stages of the web programme as there was a higher dropout at stage 2. This component of the web programme was simplified based on participant feedback. Both the length of time to complete stage 2 (aerobic training) and 3 (strength and aerobic training) were shortened and completion rates improved. Similar numbers withdrew after this modification; however, these were due to exacerbations, other problems from comorbidities and technology problems meaning patients did not even register or come for their introduction. Although more patients withdrew from the web arm of the study (12 from the PR group vs 29 from the web group), there were no significant differences between the groups in any outcome; those that did complete the website, as well as those that had completed conventional PR.

Priorities around the use of technology within the NHS are changing^4^ but this will not be without its challenges. A previous evaluation to explore the use of technology within a COPD population which was carried out by our institution found although patients owned a computer or mobile phone, usage was limited and was predominantly within the younger age range.^40^ Seventeen per cent of interested participants in this study did not have access to the internet and highlights the need to assess access as well as the competency of patients being able to use the web prior to starting the web programme. The majority of patients that entered the study expressed a preference for randomisation to the website arm of the study, showing that there is a desire for this type of intervention. Although a greater proportion of patients withdrew from the classes, stating that they would have preferred the web-based programme, it is not known if they would have engaged with and completed the programme. The study showed a lack of engagement in technology in this particular population despite a great deal of patient-user involvement in the site development.

The trial design meant that patients needed to be willing to be randomised to either group, whereas in clinical practice it is more likely that patients will have a preference due to genuine choice or practical difficulties that precludes access to supervised rehabilitation programmes. This patient choice or preference may improve uptake and completion of a PR programme. By exploring alternative forms, such as a web-based programme, patients that potentially would decline standard PR are provided with an alternative form of intervention. Alternative formats of CR, including home-based CR such as the Heart Manual and the Angina Plan,^41^ have been shown to be an effective alternative to conventional CR. In both randomisation groups, when patients were asked, the highest per cent stated their preference was the web programme. Those randomised to the conventional PR classes who were less disabled (MRC 2) and younger would have preferred the web programme, whereas the older patients preferred to attend the classes. Studies have shown that most patients with coronary heart disease who are still working prefer to follow a home-based rehab programme instead of conventional supervised classes.^42^

Web-based rehabilitation may inform the design of future trials. The data collected in this study appear to have a role in the delivery of PR. However, a stratified approach may be needed based on patient need and choice of delivery to achieve the best outcomes for patients and deliver a cost-effective model of rehabilitation for a wider population.
