# Supplementary material for: Interactive web-based pulmonary rehabilitation programme: a randomised controlled feasibility trial
Source: BMJ Open. 2017 Mar 31;7(3):e013682. doi: 10.1136/bmjopen-2016-013682 (PMC5387978; doi:10.1136/bmjopen-2016-013682)
Supplement: supplementary material [file bmjopen-2016-013682supp.pdf]

Supplement for the Web paper

Stages of the WEBsite

SPACEforCOPD® (Figure 1) is an interactive Web-based PR programme that offers a comprehensive package of exercise and education. It was developed following the success of Activate Your Heart which was devised by cardiac rehabilitation specialists at our institution, UHL NHS trust. The programme is password protected; each participant was given their own unique password to access the SPACEforCOPD® programme. All participants were able to record and monitor their exercises and strength training as well as interacting with members of the research team (Figure 2).

The SPACEforCOPD® programme was structured to guide the user through four stages that each have specific tasks the user needs to achieve before progressing onto the next stage (Figure 3). Tasks included creating and updating their own short-term goals, completing knowledge tests on COPD and exercising safely, and reading specific topics such as inhaler techniques. The educational reading material includes videos and covers topics such as disease education, managing breathlessness, recognising the signs and symptoms of an exacerbation, energy conservation and diet and healthy eating (Figure 4).

In Stage 1, participants were asked to do a multiple choice questionnaire to establish their knowledge regarding the principles of exercising safely. A score of 80% was set as a threshold to ensure understanding of these principles. In Stages 2-4, participants were required to record all their exercises, both aerobic and strength, in an exercise diary. During Stage 2, participants were advised to record their aerobic exercise, 5 days out of 7. The intensity of the exercise was based on their performance on the baseline ISWT and ESWT exercise tests and prescribed at 85% of baseline performance. In Stage 3, as well as completing 5 out of 7 days of aerobic exercise, patients were asked to also start their resistance training, completing 3 strength sessions in a week. Finally, in Stage 4, participants were required to maintain both the aerobic and strength components of the exercise programme for a further 2 weeks. There was also interactivity around stress management, knowledge and smoking cessation, if appropriate. For smokers, a cost calculator was developed that would calculate how much the user had spent or saved since starting the programme. This was delivered along with advice and support to stop smoking.

Other features embedded within the SPACEforCOPD® programme included a forum where patients were also able to share views and experiences with other programme users, a blog, and a frequently asked questions section. The forum was monitored and moderated, as necessary, by the research team. In addition patients were able to communicate privately with the research team via the Ask the Expert messaging facility.

The research team members were provided with individual passwords to access the administration section of the programme; this allowed them to view and monitor individual patients' progress and view patient login data. Patients' were contacted once a week via email or phone to discuss how they were progressing, any issues or barriers to them not continuing the programme.

All data captured on the programme were encrypted to safeguard patient confidentiality.

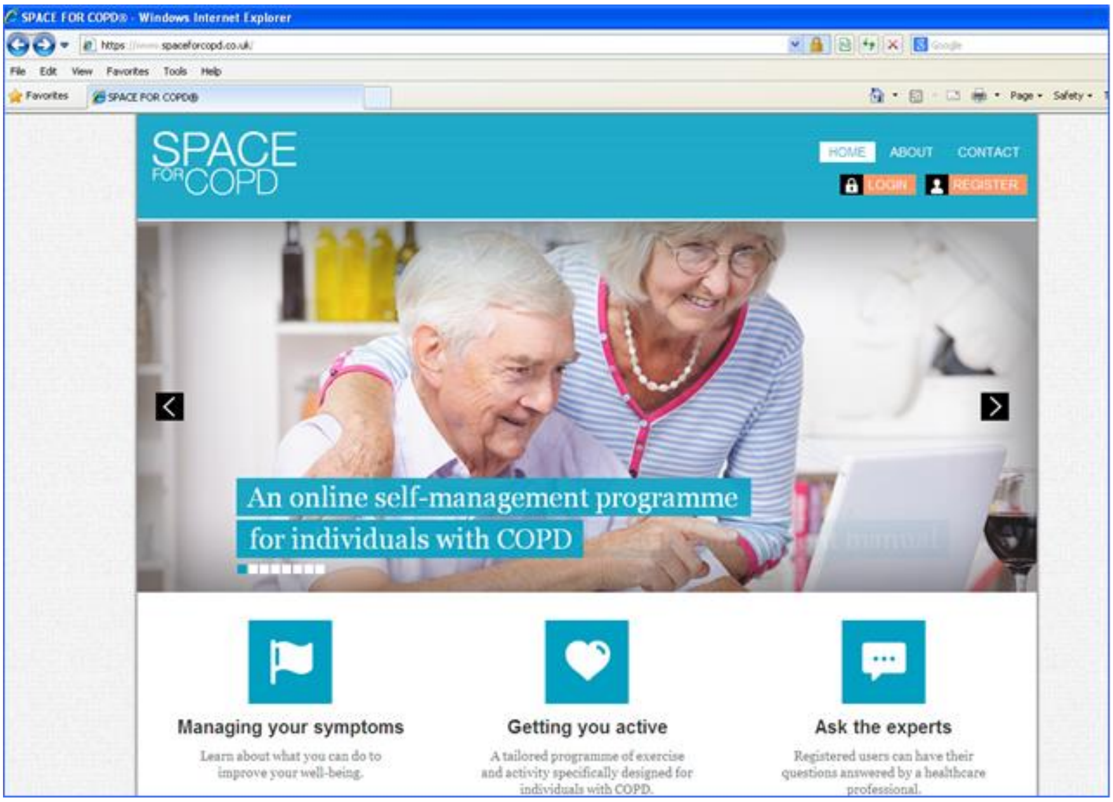

Figure 1. SPACE for COPD website homepage

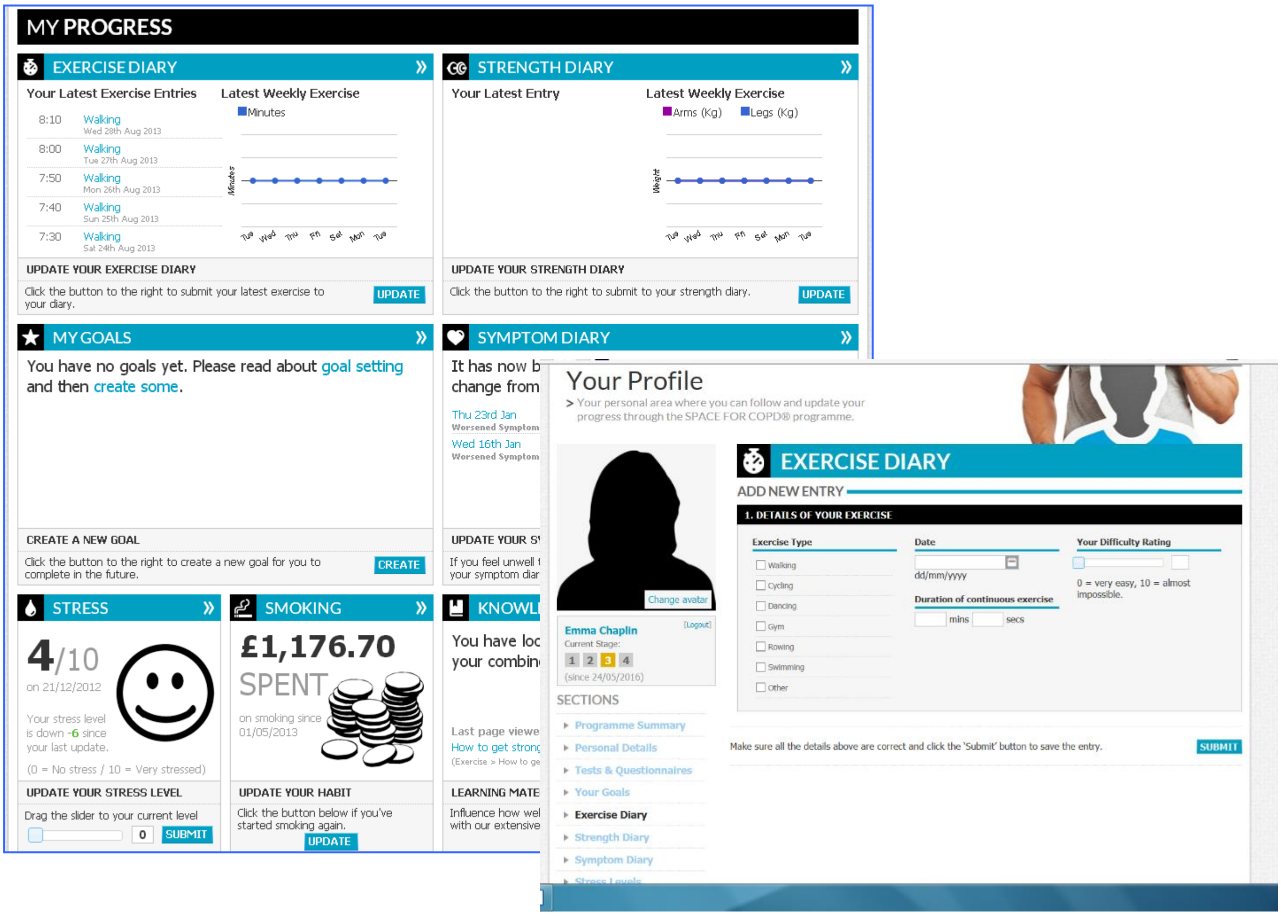

Figure 2. Recording details of exercise and strength training

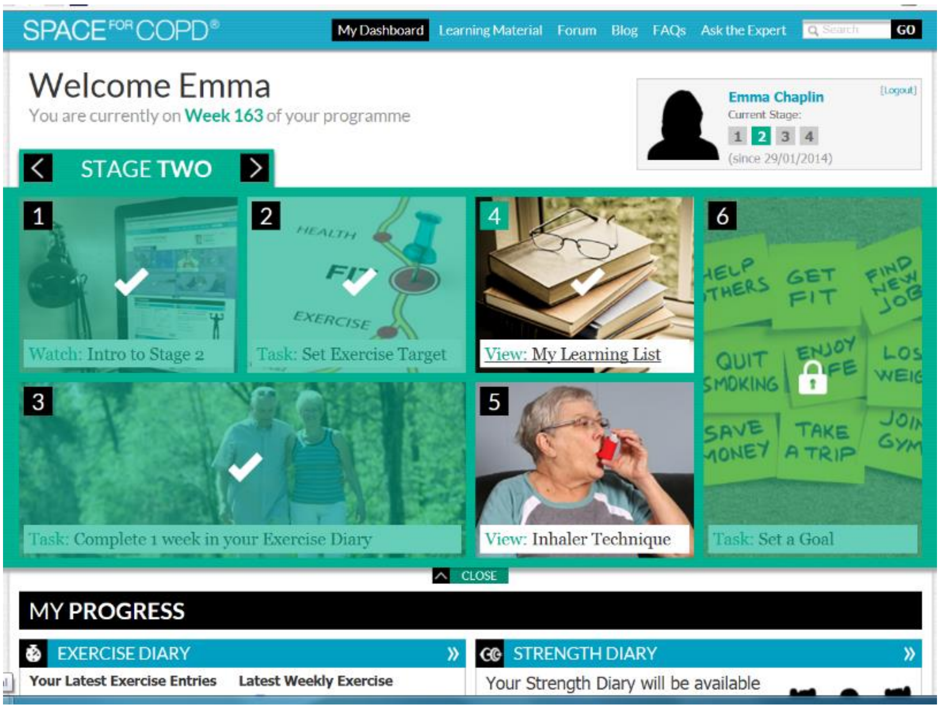

Figure 3. Part of the dashboard showing what tasks need to be completed in Stage 2

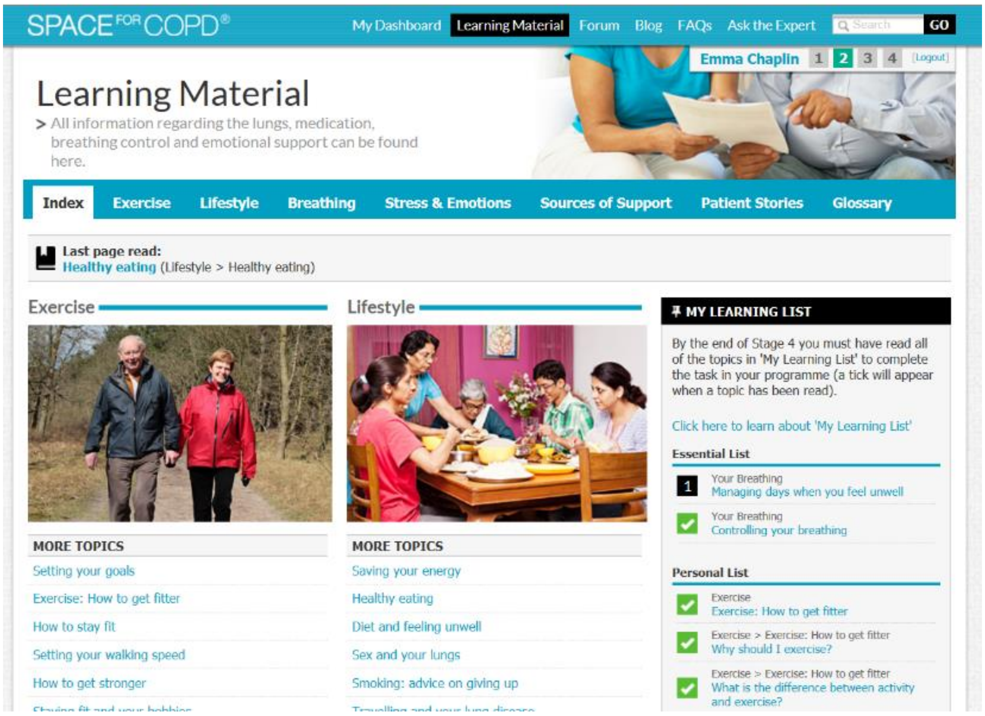

Figure 4. Reading material section
